# Linking Aromatic Hydroxy Metabolic Functionalization of Drug Molecules to Structure and Pharmacologic Activity

**DOI:** 10.3390/molecules23092119

**Published:** 2018-08-23

**Authors:** Babiker M. El-Haj, Samrein B. M. Ahmed, Mousa A. Garawi, Heyam S. Ali

**Affiliations:** 1 Department of Pharmaceutical Sciences, College of Pharmacy, Ajman University, Ajman, UAE; m.qarawi@ajman.ac.ae; 2 College of Medicine, Sharjah Institute for Medical Research, University of Sharjah, Sharjah, UAE; samahmed@sharjah.ac.ae; 3 Department of Pharmaceutics, Dubai Pharmacy College, Dubai, UAE; heyam57@hotmail.com

**Keywords:** aromatic hydroxy metabolites, arenolic drug metabolites, metabolic *O*-dealkylation, metabolic aromatic-ring hydroxylation, primary and auxiliary pharmacophores, auxophores, metabolic modification of drug activity

## Abstract

Drug functionalization through the formation of hydrophilic groups is the norm in the phase I metabolism of drugs for the modification of drug action. The reactions involved are mainly oxidative, catalyzed mostly by cytochrome P450 (CYP) isoenzymes. The benzene ring, whether phenyl or fused with other rings, is the most common hydrophobic pharmacophoric moiety in drug molecules. On the other hand, the alkoxy group (mainly methoxy) bonded to the benzene ring assumes an important and sometimes essential pharmacophoric status in some drug classes. Upon metabolic oxidation, both moieties, i.e., the benzene ring and the alkoxy group, produce hydroxy groups; the products are arenolic in nature. Through a pharmacokinetic effect, the hydroxy group enhances the water solubility and elimination of the metabolite with the consequent termination of drug action. However, through hydrogen bonding, the hydroxy group may modify the pharmacodynamics of the interaction of the metabolite with the site of parent drug action (i.e., the receptor). Accordingly, the expected pharmacologic outcome will be enhancement, retention, attenuation, or loss of activity of the metabolite relative to the parent drug. All the above issues are presented and discussed in this review using selected members of different classes of drugs with inferences regarding mechanisms, drug design, and drug development.

## 1. Introduction

Phase I metabolism of drugs (also known as the non-synthetic or functionalization phase) is mainly brought about by microsomal enzymes adding hydrophilicity to hydrophobic moieties in drug molecules. In most cases, this is attained metabolically by revealing or introducing the hydrophilic hydrogen-bonding hydroxyl group. Generally, metabolic interference may take place at pharmacophoric and/or auxophoric groups. Accordingly, the location in a drug molecule at which the metabolic change takes place may determine the pharmacologic outcome of the metabolite.

In any case, the resulting drug metabolite may be pharmacologically inactive, less active, equiactive, or even more active with respect to the parent drug. In other cases, an inactive parent drug is metabolically converted to the active form, in which case the inactive parent drug is known as a “prodrug”. Cases where the metabolic changes are intermediary in drug activation are also known.

For some drug metabolites, the pharmacologic outcomes—pharmacodynamic, pharmacokinetic, and toxicologic—prove to be substantially more favorable compared to their parent drugs. In such cases, the metabolites were developed into drugs of their own rights. In this review, such drug metabolites are referred to as “metabolite drugs”.

Generally, in drug development, the pharmacophoric or auxophoric roles of functional groups in lead drug compounds or drug prototypes are modified in the synthetic laboratory in some way. The introduction of a group in the framework, the reduction of a group to framework status, or the replacement of a group by another are the main strategies followed. Through biotransformation, the body may play an analogous in vivo role by changing pharmacophoric and/or auxophoric groups into others, or by introducing groups at certain positions of the drug molecule with concomitant changes in pharmacodynamic and/or pharmacokinetic properties of the metabolite relative to the parent drug. Such metabolic changes of a drug molecule may lead to a variety of pharmacologic outcomes. The dependence of the metabolic change on the structure of the drug molecule and the pharmacologic outcomes of the resulting metabolites are the subjects of this review.

In its interactions with drugs, the body discriminates between different chemical structures, as well as between stereoisomers of the same drug. In stereoisomers of the same drug, the atoms and groups have the same connectivity but differ in spatial arrangement, i.e., three-dimensional (3D) structures. Drug molecules that contain chiral centers exist as stereoisomers known as enantiomers. Enantiomers are designated in two ways: (a) as *R* or *S*, depending on the atomic numbers of the atoms bonded to the chiral center, or (b) as *dextro* (+) or *levo* (−) depending on the direction in which they rotate the plane of polarized light. Enantiomers of a chiral drug have identical chemical and physical properties; however, they differ in their pharmacodynamic and/or pharmacokinetic properties when they interact with the chiral environment of the body [1]. Where applicable, the pharmacodynamic differences of enantiomers are highlighted for the chiral drugs and their metabolites, which are presented as examples in this review as classes of drugs or as individual drugs. 

## 2. Objective of the Review

The objective of this review is to investigate the relationship between the metabolic change and the modification of pharmacodynamic and/or pharmacokinetic properties of drugs in an endeavor to explain when the pharmacologic activity of the metabolite is retained, decreased, lost, or even enhanced with respect to the parent drug. The link between the metabolic functionalization and pharmacologic activity to the structure of the drug is also of prime interest. Such information, as described above, will be useful in drug design when contemplating new drug entities. Additionally, it should be useful in drug development when considering what chemical changes are required in a lead drug compound or prototype for better drug efficacy and metabolic stability.

## 3. Methodology

The inclusion criteria of the selection of drugs to be reviewed include the following:

(a) Drugs that are metabolized by *O*-dealkylation or aromatic ring hydroxylation;
(b) Availability of data regarding the pharmacologic activity of the major metabolite(s).

The sources of information include the following:

- The published literature;
- Drug manufacturers’ data sheets;
- Reference books on drug metabolism and activity of metabolites.

The selection of the case-study drugs was based on varying the pharmacologic and chemical classes, as described in each section.

## 4. Review Strategy

Although the selected drugs are mostly metabolized by multi routes, only metabolic oxidative reactions that result in one chemical functionality, the aromatic hydroxy group, were considered. The representative drugs were selected based on both chemical structure and pharmacologic action. Each study/metabolite(s) case is presented in a figure including the following information subject to availability in the literature:

- The chemical structure of the drug and metabolite(s), and the enzymes or isoenzymes involved;
- The status of the pharmacologic activity of the metabolite;
- The percentage concentration of the metabolite(s) with respect to the parent drug.

Each parent drug is briefly reviewed. The arguments for the links between metabolic changes and pharmacologic activity and structure are discussed for some drug/metabolite cases, as well as being presented in an overall discussion given for all the cases at the end of the section.

## 5. Aromatic Hydroxy (Arenolic) Metabolites

Arenolic metabolites can result from one of two metabolic reactions: *O*-dealkylation of aralkoxy groups or hydroxylation of aromatic rings (mainly benzene); in the latter case, the positions of the hydroxy groups are determined by the prevailing electronic and steric effects on the aromatic ring. The benzene ring may be present in the drug molecule as a separate entity (i.e., as a phenyl group), or it may be fused to another benzene ring, or to an alicyclic or a heterocyclic ring. When the benzene ring is fused to either an alicyclic or heterocyclic ring, the resulting ring system may be described as benzenoid. Arenolic metabolites are presented and discussed in Section 5.1 and Section 5.2. 

### 5.1. Arenolic Metabolites Resulting from the O-Dealkylation of Aralkoxy Groups

**Figure 1:**
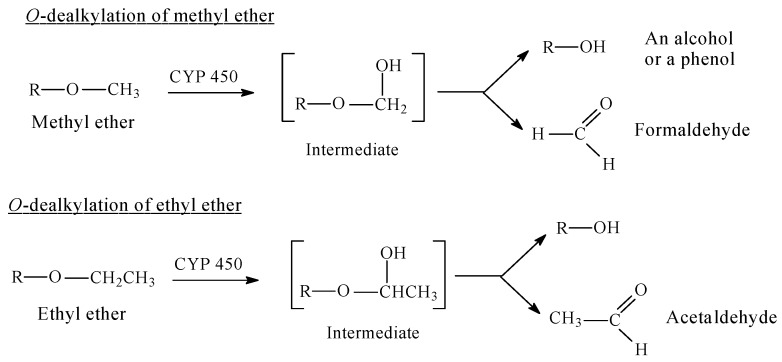
Cytochrome P450 (CYP)-catalyzed *O*-dealkylation of alkyl or aralkyl ethers.

The metabolic cytochrome P450 (CYP)-catalyzed *O*-dealkylation of aralkoxy groups involves, as a first step, the hydroxylation of the carbon atom of the alkyl group that is linked to the oxygen atom. This hydroxylated metabolite is unstable; it breaks spontaneously into two molecules: the dealkylated metabolite (e.g., an alcohol or a phenol) and an aldehyde (e.g., formaldehyde after demethylation, acetaldehyde after deethylation, etc.) [2]. The reaction is shown for a general case in Figure 1, where the R group can be aliphatic or aromatic.

The *O*-dealkylation of aralkoxy groups results in the formation of arenolic metabolites of varying pharmacologic activities, as is reviewed for the following selected cases:

(1) Enhancement of activity occurring in the opioid narcotic/analgesic drug, codeine, and its semisynthetic and synthetic congeners, all containing an aryl-methoxy group, and the analgesic/antipyretic drug, phenacetin, which contains an aryl-ethoxy group;
(2) Retention of activity by venlafaxine, (a selective-serotonin-reuptake-inhibitor (SSRI) antidepressant, containing an aryl-methoxy group);
(3) Attenuation or loss of activity by COX1/COX2-inhibitors, -naproxen, indomethacin, and nabumetone-each containing an aryl-methoxy group.

#### 5.1.1. Metabolic Aralkoxy Group Cleavage Resulting in the Enhancement of Pharmacologic Activity: Opioids and Phenacetin

##### Opioids

Opioid drugs can be defined as those acting on the various types of opioid receptors (mu, kappa, and delta) to produce a range of biologic effects. Mu-receptor stimulation is associated with analgesic (antinociceptive) effects, respiratory depression, reduced gastrointestinal motility (constipation), and euphoric and dependence effects, while kappa-receptor stimulation is associated with analgesia, psychomimetic effects, dysphoria, and diuresis [3,4].

**Figure 2:**
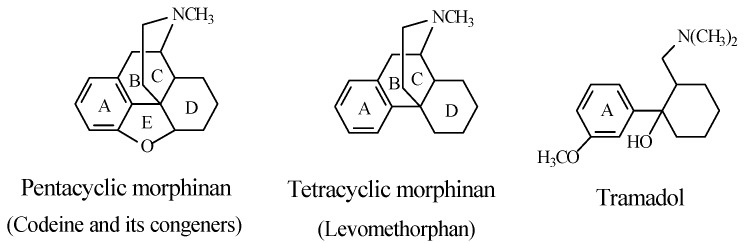
Chemical classification of methoxy-group-containing morphinan and non-morphinan opioids.

For the sake of discussion, methoxy-group-containing opioid drugs can be classified with regards to their source and chemical build-up. Source includes natural origin (e.g., codeine), semisynthetic (e.g., codeine congeners), and synthetic (e.g., tramadol). Tramadol is considered to be a synthetic codeine congener since it was developed using codeine as template. Chemically, codeine and its semisynthetic congeners are classified as pentacyclic or tetracyclic morphinans (Figure 2). On the other hand, tramadol is a non-morphinan bicyclic opioid devoid of rings B and C, as shown in Figure 2.

(1) Codeine

**Figure 3:**
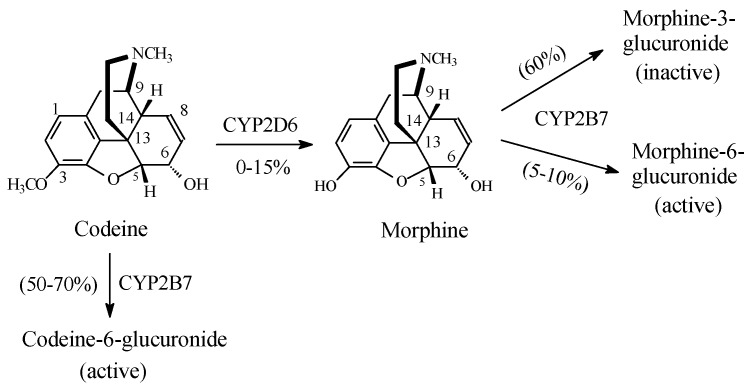
Metabolic pathways of codeine.

Codeine (Figure 3) is an opium alkaloid; it is the methyl ether of morphine and is present in opium in a 1–3% concentration [5]. It is obtained semisynthetically on a large scale by the methylation of morphine. Codeine is a weak mu-receptor agonist, being only about 0.1% as active as morphine [6]. The analgesic activity of codeine is mainly attributed to its metabolites. Codeine is metabolized as per the pathways shown in Figure 3 to codeine-6-glucuronide, morphine, and norcodeine. The resulting morphine is further metabolized to the 3- and 6-glucuronide conjugates [7,8]. As indicated in Figure 3, reports in the literature on the concentrations of codeine metabolites are variable. Further, reports on the mu-receptor affinity and analgesic activity of codeine metabolites are even contradictory. It is a longstanding belief that the mu-receptor affinity and analgesic activity of codeine are mediated through its metabolite, morphine, which has an affinity for the mu-opioid receptor 200-fold greater than that of codeine [9]. The analgesic activity of codeine was suggested to be largely due its metabolite morphine [9,10,11,12]. However, of late, Vree et al. (2000) developed a different view of codeine’s affinity to the mu-opioid receptor and its analgesic activity. The authors argued that the analgesic activity of codeine was due to its glucuronide conjugate rather than to its *O*-desmethyl metabolite, morphine [13].

The literature’s contradictory evidence on the mu-receptor affinity and the analgesic activity of codeine and its metabolites are weighed in the discussion section.

It may be of interest to note that codeine’s metabolic pathways cover almost the entire spectrum of biological activity, including reactions that are augmenting, activating, inactivating, and attenuating of the analgesic activity of the metabolite with respect to the parent drug.

(2) Codeine Congeners

**Figure 4:**
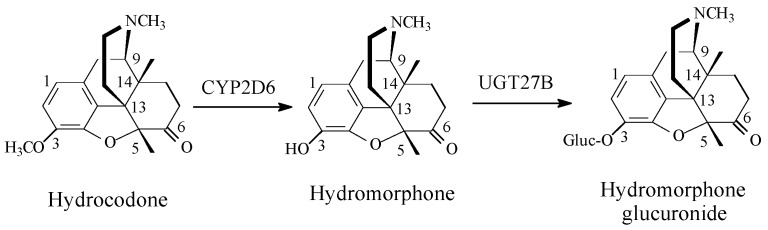
Metabolic pathways of hydrocodone.

**Figure 5:**
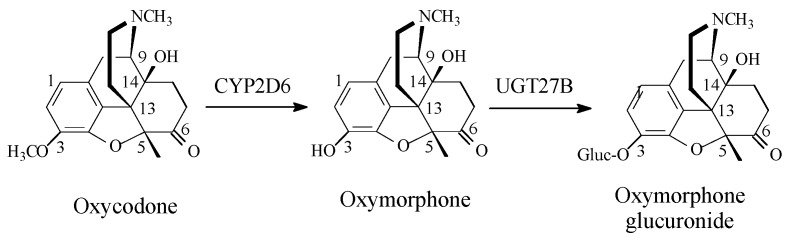
Metabolic pathways of oxycodone.

**Figure 6:**
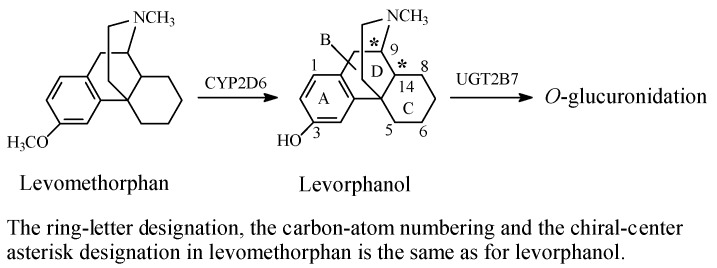
Metabolic pathways of levomethorphan.

**Figure 7:**
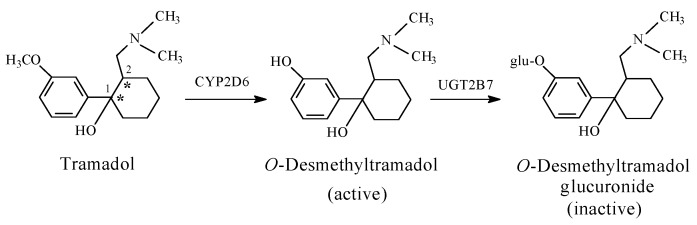
Metabolic pathways of tramadol.

Codeine synthetic pentacyclic-morphinan congeners include hydrocodone, oxycodone, and the tetracyclic morphinan congener, levomethorphan, which all have 3-arylmethoxy groups Figure 4, Figure 5 and Figure 6. On the other hand, tramadol (Figure 6) may be considered a non-morphinan codeine analog since its development was based on codeine as template. Tramadol contains an arylmethoxy group in a position reminiscent to that of codeine. While hydrocodone, oxycodone, and tramadol are currently clinically used in pain management [14,15], levomethorphan has never been used. The four drugs are metabolized by the isozyme CYP2D6 to their corresponding *O*-desmethyl phenolic counterparts, as shown in the corresponding Figure 4, Figure 5, Figure 6 and Figure 7 [16,17,18]. Due to their substantially higher mu-receptor affinities, and accordingly, their higher analgesic activities compared to their parent drugs [15,16], the four phenolic metabolites were developed into drugs of their own rights. Presumably, the increase in mu-receptor affinity and analgesic activity is a result of the phenolic hydroxy groups in the four-metabolite drugs. We present a possible explanation of why this is the case in the discussion section. 

The subject of stereochemistry is of paramount importance when considering drug structure–activity relationship of chiral drugs. Of the four codeine congeners, tramadol and levorphanol present the most interesting features. 

##### Stereochemistry of the Morphinan Opioids

The stereochemistry of the pentacyclic morphinan opiates, to which belong morphine and codeine, is complicated by their rigid ring system and the presence of multi-chiral centers. Morphine and codeine both contain five chiral centers, as indicated by the wedged bonds in Figure 3. Assigning absolute configuration to each chiral center and then an overall absolute configuration to either a codeine or a morphine molecule is a challenge. With a total number of 32 enantiomers, the task is overwhelming. However, for the purpose of designation of chirality, optical activity may be used. In multi-chiral center molecules, optical activity is an additive function of the rotations at the component asymmetric centers. In morphine, the most important member of the pentacyclic morphinans, the chiral centers at C5, C6, and C9 (Figure 3) rotate the plane of polarized light to the left (*levo* (−)) while the remaining centers at C13 and C14 do so to the right (*dextro* (+)) [19]. Depending on the overall optical rotation, the analgesic activity of natural morphine is attributed to levomorphine [20]. 

As shown in Figure 6, the tetracyclic morphinan, levorphanol, has two chiral centers at C9 and C14, in addition to exhibiting *cis*/*trans* isomerism. Despite the fact that the B/C *cis* (14*R*) configuration is two-fold less active than the B/C *trans* isomer, it is the one that is clinically used as a narcotic analgesic [21]. The additive optical rotation of the plane of polarized light of the two chiral centers in levorphanol must be to the left, thus accounting for the origin of the prefix *levo*. The fact that enantiomers of chiral drugs may have different actions is demonstrated by the *dextro* counterpart of levorphanol, i.e., dextrophan, which is used as an antitussive, and is devoid of the narcotic analgesic activity characteristic of the *levo* enantiomer [22].

The non-morphinan opioid drug, tramadol, contains two chiral centers, as indicated by asterisks in Figure 7, and accordingly, it exists in four enantiomeric forms. In addition, because it contains a cyclohexane ring, tramadol exists as *cis*/*trans* isomers. Despite the fact that tramadol is marketed as the racemate of the *cis* isomer, its enantiomers and active *O*-desmethyl metabolite have significant selectivity in their analgesic mechanism of action. Specifically, the 1*S*,2*S*-(−) enantiomer inhibits norepinephrine reuptake, while the 1*R*,2*R*-(−) enantiomer inhibits 5-HT (serotonin) reuptake to produce analgesic activity. On the other hand, the dextrorotatory *O*-desmethyltramadol produces a six-fold greater analgesic activity than tramadol via stimulating the mu receptors [23]. 

##### Discussion of Opioids

The metabolic *O*-demethylation of the methoxy-group-containing opioids is discussed separately because of the availability of substantial supportive evidence.

A number of theories were proposed to account for the role of the phenolic–hydroxy group in mu-receptor interaction and the consequent production of analgesia.

**Figure 8:**
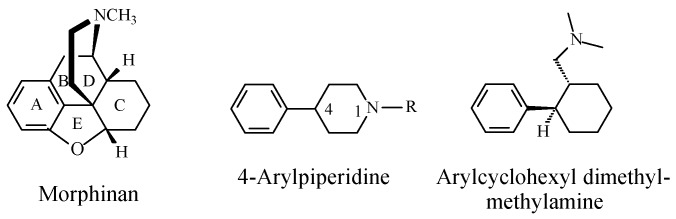
Structures of the opioid drug pharmacophores.

When discussing the structure–activity relationship of opioids, Foye (2013) [24] classified opioid drugs into two categories according to the pharmacophore responsible for mu-receptor interaction: the rigid multicyclic (morphinan) opioids (exemplified by morphine, codeine, and codeine congeners), and the flexible opioids (exemplified by 4-arylpyridinepethidine). To the latter category, we may add arylcyclohexylmethylamine, which is found in tramadol. The structures of the three categories are depicted in Figure 8. 

Foye (2013) [24] argued the importance of a phenolic–hydroxy group on ring A (Figure 8) to the mu-receptor interaction and analgesic activity of the rigid multicyclic morphinan opioids such as morphine and the *O*-desmethyl metabolites of codeine congeners. The author also maintained that a phenolic–hydroxy group is not a requirement for the mu-receptor interaction and analgesic activity of the flexible non-morphinan opioids. It should be noted that none of the latter groups of drugs contains a phenolic hydroxy group and that aromatic-ring hydroxylation is not reported as a metabolic route for any of them.

**Figure 9:**
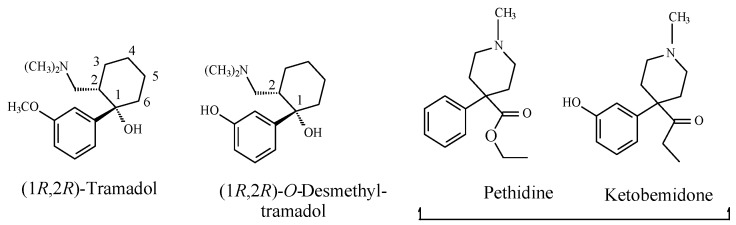
Tramadol/*O*-desmethyltramadol and pethidine/ketobemidone.

Foye’s theory of “phenolic OH binding liability” for the flexible opioids [24] may be contested based on observations from the metabolic activity of *O*-desmethyltramadol (a metabolite of tramadol; Figure 7) and ketobemidone (an analog of pethidine; Figure 9). Both drugs can be categorized as non-rigid flexible non-morphinan opioids. *O*-Desmethyltramadol exhibits a 200-fold increase in mu-receptor affinity and analgesic activity relevant to tramadol [25]. Ketobemidone, on the other hand, has a four-fold mu-receptor affinity and analgesic activity compared to pethidine [26]. The enhanced mu-receptor affinity and analgesic activity of both *O*-desmethyltramadol and ketobemidone can be attributed to the phenolic hydroxy group, which is in a position reminiscent to that of morphine.

Another opioid drug–receptor interaction theory was suggested by Beckett and Casy (1959) [27] who stated that the macromolecule with which the analgesic interacts has, or attains, a certain conformation into which the phenolic group must fit before the biological effect (of analgesia) can occur. A “three-point” attachment of the macromolecule to a substrate’s flat aromatic moiety, its basic center, and its hydrocarbon area was postulated.

Glucuronide and acetyl derivatives of morphine provide substantiating evidence of the phenolic hydroxy group’s involvement in strengthening the mu-receptor affinity, and accordingly, the analgesic activity of the morphinan-opioid drugs that contain it, either intrinsically or metabolically produced. Further discussion of this point follows. 

By virtue of its phenolic–hydroxy group at position 3 and alcoholic hydroxy group at position 6, morphine (Figure 3) forms two glucuronide conjugates: morphine-3-glucuronide and morphine-6-glucuronide (Figure 3). While morphine-6-glucuromide is a far more potent mu-receptor agonist and analgesic than morphine [28,29,30], morphine-3-glucuronide is devoid of both effects [31]. Furthermore, the *O*-glucuronide conjugates of both *O*-desmethyltramadol (Figure 6) and levorphanol (Figure 7) are devoid of mu-receptor agonistic effects, and accordingly, of analgesic activity [32,33]. The above data may be explained based on size and hydrogen-bonding ability differences between the hydroxy and glucuronide groups. In morphine, the hydrogen bonding provided by the 6-OH group is important for mu-receptor fitting, and thus, analgesic activity. With three hydroxy moieties, the glucuronide group at position 6 of morphine is capable of establishing more hydrogen bonds than the 6-OH. Further stronger interactions of the glucuronide group with the mu receptor involve ion–ion and ion–dipole binding provided by the carboxylate (COO^−^) moiety. Such a state of affairs will most probably lead to stronger mu-receptor fit, and hence, higher analgesic activity of morphine-6-glucuronide than morphine. On the other hand, the size (steric) factor favors the hydroxy group at position 3 of morphine to anchor the aromatic ring in its hydrophobic pocket in the mu receptor, rather than the considerably bigger glucuronide group. 

**Figure 10:**
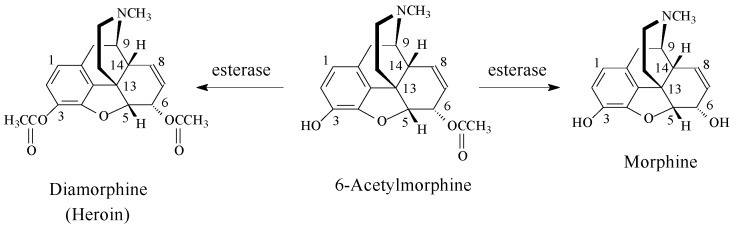
Diamorphine, 6-acetylmorphine, and morphine.

Upon comparing the mu-receptor affinity and analgesic activity of morphine, 6-acetylmorphine, and diamorphine (heroin) (Figure 10), 6-acetylmorphine was found to be the most active of the three opiates; it is four times as active as morphine. Heroin is also more active than morphine by a factor of two, but less active than 6-acetylmorphine [34,35].

A plausible explanation of the above data is as follows: being more lipophilic than morphine, both 6-acetylmorphine and heroin (Figure 10) will cross the blood–brain barrier faster and in higher concentrations than morphine. On the other hand, having a free phenolic hydroxy group, 6-acetylmorphine will interact with the opioid mu receptor more efficiently than diamorphine. In diamorphine, the free hydroxy group is generated metabolically in the brain by esterase hydrolysis, which will lead to a delayed effect and reduced efficacy. The effects of morphine and the two acetylated opiates (diamorphine and 6-acetylmorphine) can, therefore, be explained by a combination of pharmacodynamic and pharmacokinetic influences.

Additional substantiating evidence for the role of the free phenolic hydroxy group may be obtained from levomethorphan and its *O*-desmethyl metabolite, levorphanol (Figure 7). These two tetracyclic-morphinan opiate drugs lack the 6-hydroxy group, and hence, the ability to form glucuronide conjugates at that position; yet, levorphanol has a stronger affinity for the mu receptor and analgesic activity than its parent drug, levomethorphan [36].

In conclusion, we may summarize the role of the hydroxy group in arenolic opioids in the following statement: as shown in the opioid pharmacophores in Figure 8, the aromatic rings labeled A, present in both morphinan and non-morphinan opioids, are essential components of the pharmacophore of the opioid–mu-receptor interaction. The high affinity of the arenolic opioids for the mu receptor is an indication of a logistic role played by the hydroxy group. Through hydrogen bonding with an adjacent amino-acid residue in the mu receptor, the hydroxy group plays the logistic role of anchoring the aromatic ring to the assigned hydrophobic pocket in the receptor, thus enhancing both affinity and efficacy. Substantiating evidence to the above statement is provided by the work of Sahu et al. (2008) [37] on tetrahydroimidazobenzodiazepinones (the human immunodeficiency virus 1 (HIV-1) non-nucleoside reverse transcriptase (NNRT) inhibitors). In this class of compounds, the predominant hydrophobic proper orientation for maximum effect was found to be enhanced by hydrogen bonding and polar interactions.

The assertion by Vree et al. of the analgesic activity of codeine being entirely due to its glucuronide conjugate [13] may now be reconsidered in view of the evidence so far presented on the role of the arenolic hydroxy group in opioid drugs. It is important to emphasize that the conclusion by Vree et al. was abstract rather than experimental, and as such, may be subject to a difference of opinion. 

Because both codeine and tramadol have intrinsic analgesic activities, they can be viewed as prodrugs of morphine and *O*-desmethyltramadol, respectively, both having sustained-release effects. Both morphine and *O*-desmethyltramadol are strong mu-receptor agonists used in the management of severe pain, but have the disadvantages of causing dependence and tolerance. Therefore, for the management of mild-to-moderate pain, it would be advisable to use the corresponding prodrugs, codeine and tramadol, which have the advantage of sustained release. However, some people who are poor CYP2D6 metabolizers do not make use of the beneficial prodrug sustained-release effect. For such people, it is advisable to adjust the dose of the parent drug, to administer *O*-desmethyl metabolites, or to seek alternative therapies. The frequency of the phenotype of poor metabolizers differs among ethnic groups. Less than 1% of Asians, 2–5% of African Americans, and 6–10% of Caucasians are poor metabolizers of CYP2D6 [38,39,40].

**Figure 11:**
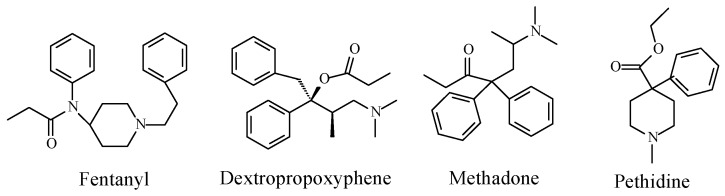
Highly hydrophobic opioids.

In addition to the pharmacodynamic receptor interactions of the methoxy opioids (occurring mainly through their polar hydroxy metabolites), the analgesic effects of hydrophobic opioid drugs, such as fentanyl, dextropropoxyphene, methadone, and pethidine (Figure 11), were mainly explained by pharmacokinetic effects. These drugs cross the blood–brain barrier more efficiently, and accordingly, they reach the mu-receptors in the brain in higher concentrations than the methoxy-group-containing members [41].

##### Acetanilide/Phenacetin/Paracetamol

**Figure 12:**
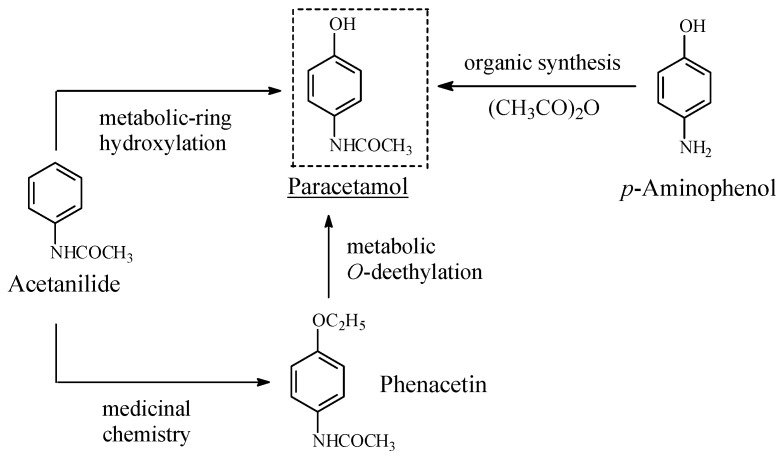
From acetanilide to phenacetin to paracetamol.

The story of the development of the most commonly used analgesic antipyretic drug, paracetamol, as a metabolite drug of acetanilide and phenacetin is depicted in Figure 12. Both the latter drugs were once used as analgesics and antipyretics. Paracetamol results from phenacetin by metabolic *O*-deethylation [42], and from acetanilide by metabolic *para*-hydroxylation of the aromatic ring [43]. Compared to its two precursors, paracetamol was found to have superior pharmacologic profiles, including those of toxicity and therapeutic index [42,43]. The free hydroxy group seems to give paracetamol the edge in inhibiting, through hydrogen-bonding interaction, two prostaglandin H2 synthases, now believed to be involved in the mechanism of action of paracetamol [44]. Moreover, the hydroxy group may play the logistic role of anchoring the aromatic ring in the proper geometric orientation in the enzyme active cavity.

#### 5.1.2. Metabolic *O*-Dealkylation of Aralkoxy Groups Resulting in Retaining Pharmacologic Activity: Venlafaxine/Desvenlafaxine 

**Figure 13:**
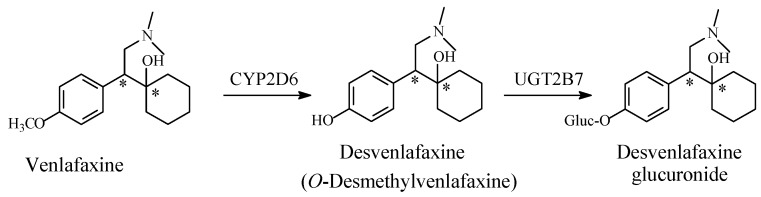
Metabolic pathway of venlafaxine.

Venlafaxine is an antidepressant in the noradrenaline–serotonin reuptake inhibitor (NSRI) category. Being chiral (Figure 13), venlafaxine exists as *R* and *S* enantiomers. Regarding neurotransmitter-reuptake inhibition, some degree of selectivity was observed: the *R-*enantiomer acted as both a noradrenaline and serotonin reuptake inhibitor, while the *S*-enantiomer acted only as a serotonin reuptake inhibitor [45]. However, the drug is marketed as the racemate. 

Venlafaxine is mainly metabolized by *O*-demethylation to equiactive desvenlafaxine (*O*-desmethylvenlafaxine; Figure 13). Venlafaxine is further metabolized through *N*-demethylation and glucuronide conjugation to inactive products (Figure 13) [46,47,48]. Desvenlafaxine was developed into a drug of its own right and approved by the Food and Drug Administration (FDA) in the United States (US) for the treatment of major depressive disorder (MDD), similar to its parent drug, venlafaxine, which is used for major depressive and anxiety disorders. However, the European Medicines Agency (EMA) had second thoughts and did not approve desvenlafaxine as a drug. They argued that venlafaxine is almost fully metabolized to desvenlafaxine, and that both compounds have essentially the same pharmacologic and pharmacokinetic profiles; hence, there is no strong reason to use desvenlafaxine as a separate drug [49]. Whether the analogy of the selective activity of the *R* and *S* enantiomers of venlafaxine may be extended to desvenlafaxine is a subject for experimental investigation. 

Venlafaxine carries structural similarity to the analgesic drug, tramadol. The latter drug exerts its analgesic effect mainly through serotonin–norepinephrine reuptake inhibition (SNRI) [50], and to a minor extent, through blocking of the mu-receptor. The *O*-Demethylation of both venlafaxine and tramadol resulted in active metabolites that were developed into drugs of their own rights. The fact that neither venlafaxine nor desvenlafaxine has analgesic effects, and that neither tramadol nor *O*-desmethyltramadol has antidepressant effects, emphasizes the close link between drug action and drug structure.

#### 5.1.3. Metabolic *O*-Dealkylation of Aralkoxy Groups Resulting in the Attenuation or Loss of Pharmacologic Activity: NSAIDs (Naproxen, Indomethacin, and Nabumetone)

NSAIDs fall into five chemical classes: arylalkanoic acids, salicylates, fenamates, oxicams (cyclic sulfonamides), and diarylheteroaromatics. The benzene ring, either as a separate entity or fused with other rings, constitutes an integral part of the pharmacophore in all the NSAID chemical classes. The carboxyl group (in the form of a carboxylate ion, COO^−^) forms the other part of the pharmacophore in the arylalkanoic acid NSAIDs. With the exception of aspirin, the mechanism of action of the NSAIDs involves the competitive inhibition of arachidonic acid, the precursor of prostaglandins, from accessing the COX active cavity. The binding of the arylalkanoic acid NSAID to the amino-acid moieties in the COX active cavity involves ion–ion, ion–dipole, and hydrogen bonding through the carboxylate group (COO^−^) and hydrophobic interactions through alkyl and aryl groups [51,52,53,54]. The alkyl groups are those of the methoxy and propionic acid moieties. 

**Figure 14:**
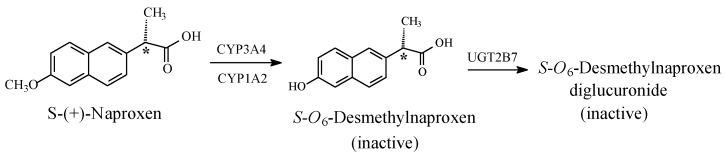
Metabolic pathway of naproxen [55]

**Figure 15:**
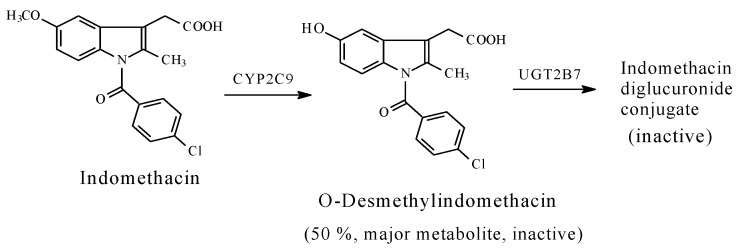
Metabolic pathway of indomethacin [56].

**Figure 16:**
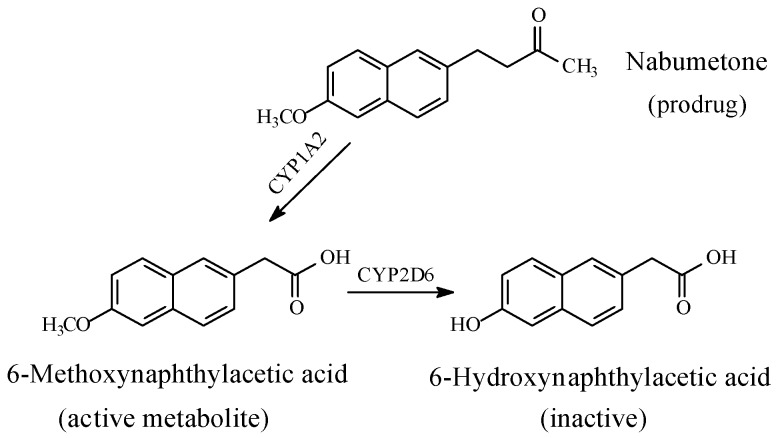
Metabolic pathways of nabumetone [57].

Naproxen, indomethacin, and nabumetone (Figure 14, Figure 15 and Figure 16, respectively) are NSAIDs that act through COX1/COX2 inhibition. They are about the only three arylalkanoic acid NSAIDs that contain aromatic methoxy groups. Furthermore, nabumetone is a prodrug NSAID, which must first be activated by the metabolic oxidation of the carbonyl group to the carboxy derivative, 6-methoxynaphthylacetic acid, as shown in Figure 16. 

##### Stereochemistry of Arylalkanoic Acid NSAIDs

The arylalkanoic acid NSAIDs presented in this section fall into two subclasses: arylpropionic acid NSAIDs, to which belongs naproxen (Figure 14), and arylacetic acid NSAIDs, to which belong indomethacin and 6-methoxynaphthylacetic acid, the active metabolites of nabumetone (Figure 15 and Figure 16, respectively). Naproxen contains a chiral center at the α-carbon of the propionic acid moiety as indicated by an asterisk in Figure 14, and so do other members of the arylpropionic acid NSAIDs, such as ibuprofen and ketoprofen. Accordingly, these drugs exist as enantiomeric pairs, *R*-(−) and *S*-(+). The COX-inhibition activity of the arylpropionic acid NSAID subclass was found to reside in its *S*-(+)-enantiomers while the *R*-(−)-enantiomers were inactive [58]. However, the inactive *R* enantiomers in some members, such as naproxen and ibuprofen, are metabolically inverted unidirectionally to the active *S*-enantiomers, and hence, may be considered as prodrugs. Generally, when the metabolic functionalization of a chiral drug occurs at a distant group from the chiral center, the absolute configuration of the enantiomers of the parent drug may be extended to its metabolite. However, this ought not to be the case with optical activity, which is only determined experimentally. Of all the arylalkanoic acid NSAIDs, only *S*-naproxen and its sodium salt are internationally marketed as enantiopure drugs. In this context, *S*-(+)-ibuprofen and *S*-(+)-ketoprofen are marketed in some countries. All the other arylpropionic acid NSAIDs are marketed as racemates. When the racemate of an enantiopure drug was either previously used or is currently in circulation, the enantiopure drug is known as a chiral-switch drug. This definition applies to *S*-(+)-naproxen whose racemate use was stopped, and to *S*-(+)-ibuprofen and *S*-(+)-ketoprofen whose racemates are currently in circulation.

*S*-Naproxen, indomethacin, and the active form of nabumetone are mainly metabolized through *O*-demethylation to give 6-*O*-desmethylnaproxen, *O*-desmethylindomethacin, and 6-hydroxynaphthylacetic acid, respectively (Figure 14, Figure 15 and Figure 16). The first two *O*-desmethyl metabolites are devoid of COX inhibitory effects, and accordingly, of NSAID activity [59,60,61]. By analogy, 6-hydroxynaphthylacetic acid, the *O*-desmethyl metabolite of nabumetone [62], is expected to be devoid of NSAID activity. According to Duggan et al. (1972), the *p*-hydroxy groups in the *O*-desmethyl metabolites place polar, hydrogen-bond-donating properties within otherwise entirely aromatic, hydrophobic, pharmacophoric groups in the parent drugs [59]. Presumably, a poor metabolite–COX fit would result in subsequent loss of pharmacologic activity. The loss of pharmacologic activity in the *O*-desmethylarylalkanoic acid NSAIDs is further reflected upon in the discussion section.

### 5.2. Overall Discussion

The phase I metabolic *O*-dealkylation of aralkoxy groups almost invariably occurs in all the drugs containing these moieties.

From the cited drug examples in this section, the metabolic *O*-dealkylation of aralkoxy groups results in three situations regarding pharmacologic activity:

(1) Enhancement of activity is exhibited by the *O*-desmethyl morphinan opioids, *O*-desmethyltramadol and *O*-desethyl phenacetin;
(2) Retention of activity is exhibited by *O*-desmethyl venlafaxine;
(3) Significant attenuation or loss of activity is exhibited by *O*-desmethyl naproxen and *O*-desmethyl indomethacin.

Some inferences can be made from the effect on pharmacologic activity in the three NSAID cases upon considering the new state of structural affairs created by the loss of the hydrophobic aralkoxy-alky group and the generation of the hydrophilic hydroxy group. The effect mostly depends on the site of drug action involved. The cases where the activity is enhanced involve the opioid drugs acting on the mu-receptor. In these drugs, the aromatic ring A, an integral part of the pharmacophore (Figure 14), binds to the receptor through hydrophobic forces of attraction. The hydroxy group on the aromatic ring plays the logistic role of optimally anchoring the hydrophobic aromatic ring in its hydrophobic pocket in the mu receptor [24]. The same theory may be extended to the acetanilide/phenacetin/paracetamol case, where the sites of action are the H2 synthase enzymes used in the formation of prostaglandin, as was recently proposed [44].

The loss of pharmacologic activity upon *O*-demethylation of the aryl-methoxy group is mainly observed in the NSAIDs, naproxen and indomethacin, and is inferred for nabumetone. In this class of arylalkanoic acid NSAIDs, COX inhibition is due to hydrogen bonding and ion pairing due to the carboxyl group, and van der Waals contacts due to hydrophobic groups, mainly the aromatic rings. In addition, in the arylpropionic acid NSAIDs, the α-methyl group forms an essential hydrophobic interacting group. Furthermore, the methyl group in the methoxy moiety in naproxen, nabumetone, and indomethacin occupies a hydrophobic pocket in COX. Duggan et al. (2010) [59] reported that the methoxy group of naproxen is oriented toward the apex of the COX active site, and forms van der Waals interactions with Trp 387 and Tyr 385. The methoxy group in naproxen (as well as in nabumetone and indomethacin) is of special importance since its metabolic demethylation results in the polar, hydrogen-bonding hydroxyl group. According to Duggan et al. (2010) [59], the hydroxyl group in *O*-desmethylnaproxen places polar, hydrogen-bonding properties within an entirely hydrophobic pocket that was occupied by the methyl group of the methoxy moiety in naproxen, which is consistent with a reduction in COX inhibition. 

Furthermore, a pharmacokinetic effect is most probably involved in the attenuation of COX inhibition by the *O*-desmethyl metabolites of naproxen, 6-methoxynaphthylacetic acid, and indomethacin. In explanation, aromatic hydroxy groups are almost invariably metabolized in phase II to the highly water-soluble and rapidly eliminated glucuronide (and sometimes sulfate) conjugates. Such an effect will lead to a reduction in the effective concentration of the metabolite at the receptor or enzyme active cavity, thus resulting in curtailing or losing activity. According to Fura (2006), attenuation or loss of pharmacologic activity is associated with the biotransformation of pharmacophoric groups and the possible accompanying changes in physicochemical properties [61].

The retention of antidepressant activity of desvenlafaxine indicates a non-essential hydrophobic binding role of the methoxy methyl group in venlafaxine. Nevertheless, a hydrogen-bonding role may not be excluded since it is provided by the methoxy oxygen in venlafaxine and by the hydroxy group in desvenlafaxine; however, this occurs more prominently in the latter drug. 

### 5.3. Arenolic Metabolites Resulting from Aromatic Ring Hydroxylation

**Figure 17:**
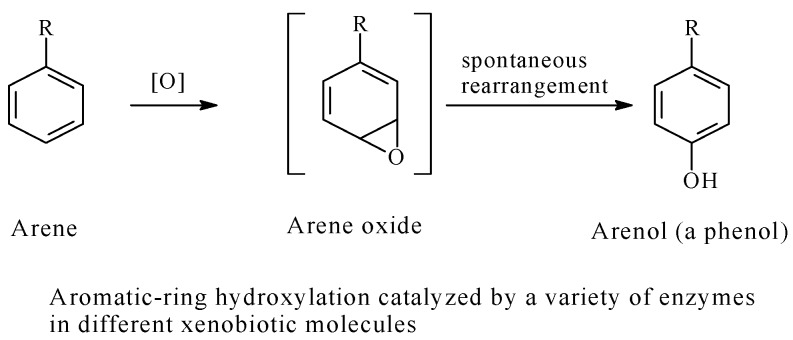
Mechanism of aromatic-ring hydroxylation.

The mechanism of metabolic aromatic-ring hydroxylation involves, as a first step, the formation of an epoxide (arene oxide) intermediate, which rearranges rapidly and spontaneously to the arenol product in most instances (Figure 17) [62,63]. 

Metabolic aromatic-ring hydroxylation assumes its importance from the fact that a large number of drug molecules contain the benzene ring as a separate entity (phenyl) or fused with other rings, alicyclic or heterocyclic. In drug molecules, aromatic rings serve the dual role of providing relatively large hydrophobic sites for interaction with receptors or acting as carriers for other functional groups. However, not all of the benzene rings in drug molecules are subject to metabolic hydroxylation. This is because certain electronic and steric effects dictate the metabolic hydroxylation of aromatic rings. These effects include the following, with possible anomalous events [64,65]:

(a) The least substituted aromatic ring will be favorably oxidized, especially at the least hindered carbon atom;
(b) The activated ring (i.e., the ring bearing an electron-donating group such as an alkyl) will be better oxidized;
(c) Ring-deactivating groups (generally groups with negative inductive effects such as halo and nitro groups) discourage ring hydroxylation;
(d) Being the farthest from steric effects in di-substituted benzene rings, the *para* position is the favored site of hydroxylation;
(e) If two aromatic rings in a drug molecule have the same chemical environment, hydroxylation will occur in only one of them;
(f) When the parent drug contains an aromatic hydroxy group, further metabolic hydroxylation is generally not favored even if there is more than one aromatic ring.

Aromatic-ring hydroxylation of drugs leads to the formation of inactive metabolites, metabolites with attenuated activity, and metabolites that are equiactive with the parent drugs. These situations are reviewed using selected representative drugs. The basis for the choice of the candidate drugs is as follows:

- Varying the chemical classes of the drugs;
- Varying the pharmacologic class of the drugs, and accordingly, the type of drug-site-of-action interaction involved (i.e., the mechanism of action of the class of drugs);
- Varying the aromatic characteristics in both the number of rings and chemical environment.

**Figure 18:**
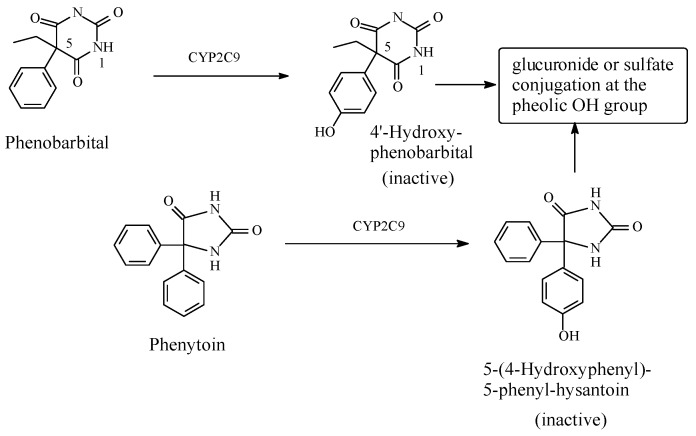
Major metabolic pathways of phenobarbital and phenytoin.

**Figure 19:**
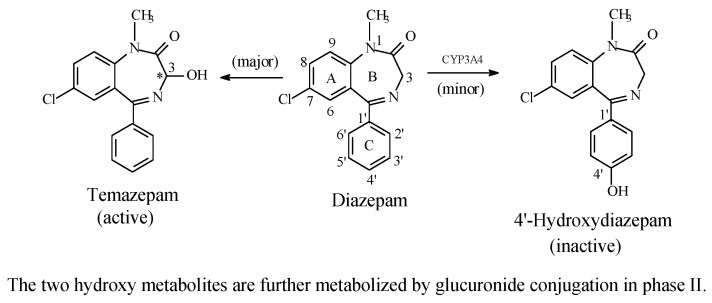
Metabolic pathways of diazepam.

**Figure 20:**
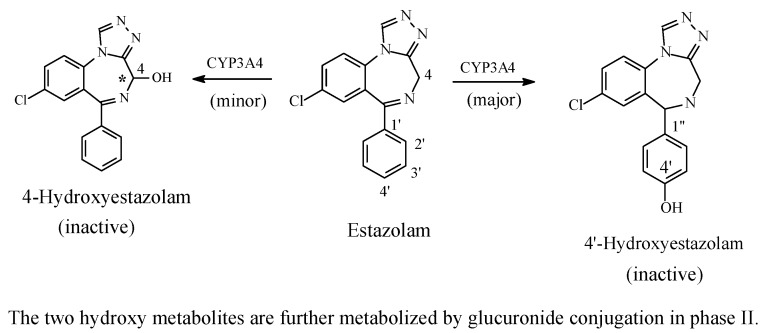
Metabolic pathways of estazolam.

**Figure 21:**
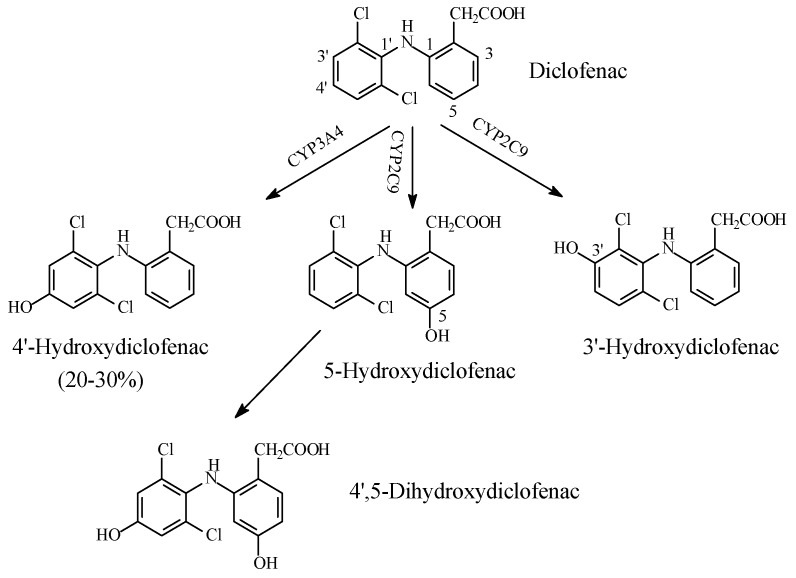
Metabolic pathways of diclofenac.

**Figure 22:**
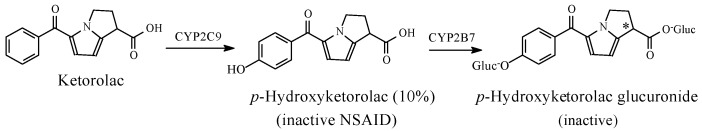
Metabolic pathways of ketorolac.

**Figure 23:**
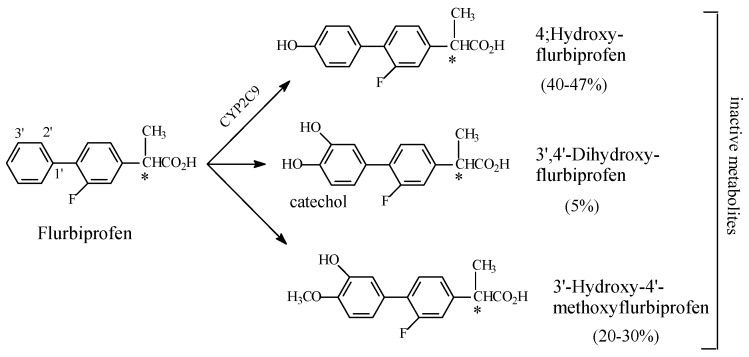
Metabolic pathways of flurbiprofen.

**Figure 24:**
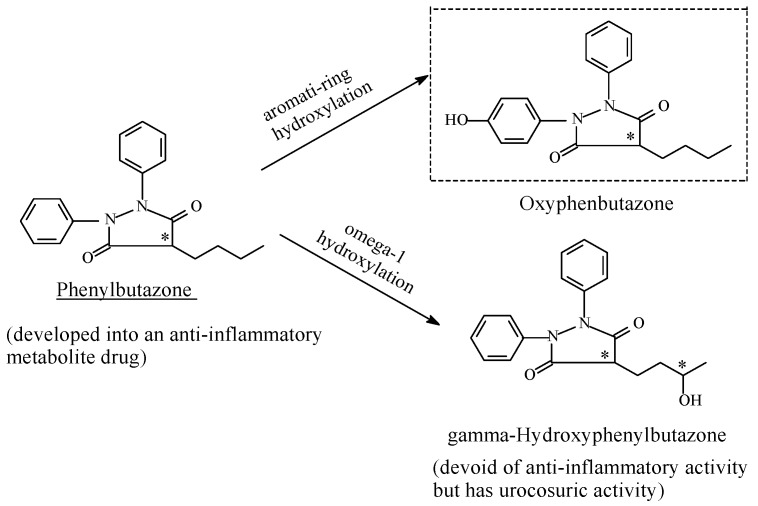
Metabolism of phenylbutazone.

**Figure 25:**
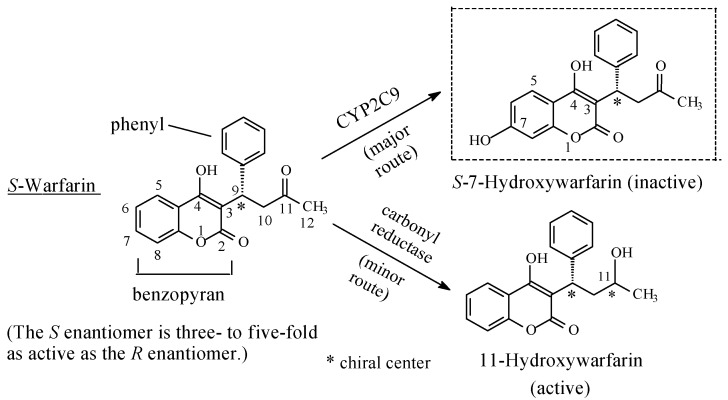
Metabolic pathways of warfarin.

Metabolic aromatic-ring hydroxylation leading to the loss of activity is exemplified by the following:

(a) The central nervous system depressant anticonvulsant drugs, phenobarbital and phenytoin, of the imide chemical class (Figure 18);
(b) The CNS depressant tranquilizer benzodiazepines, diazepam (Figure 19) and estazolam (Figure 20);
(c) The arylalkanoic acid NSAIDs, diclofenac, flurbiprofen, and ketorolac (Figure 21, Figure 22 and Figure 23, respectively), and the pyrazolone NSAID derivative, phenylbutazone (Figure 24);
(d) The anticoagulant warfarin (Figure 25).

**Figure 26:**
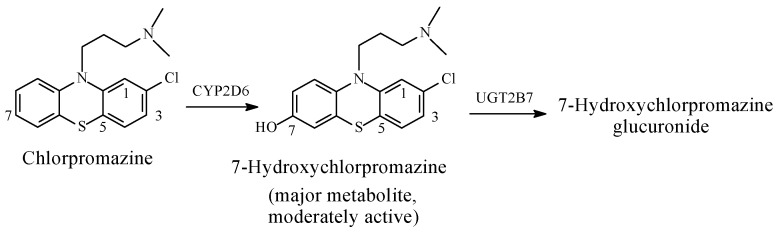
Metabolic pathways of chlorpromazine.

Metabolic aromatic-ring hydroxylation leading to attenuation of activity is exemplified by the CNS depressant and major tranquilizer, chlorpromazine (Figure 26).

**Figure 27:**
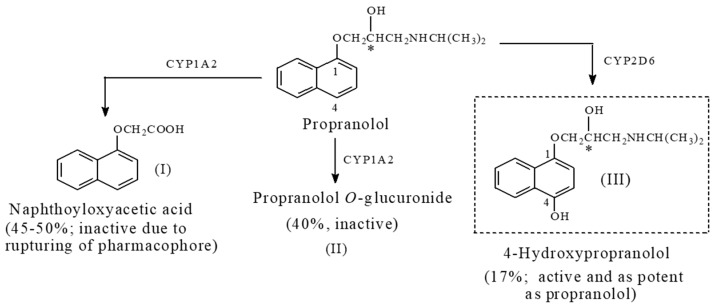
Metabolic pathways of propranolol.

**Figure 28:**
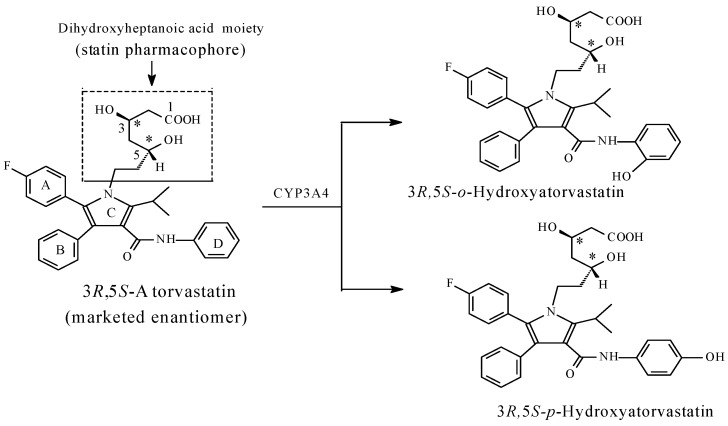
Metabolism of atorvastatin.

Metabolic aromatic-ring hydroxylation leading to the formation of equiactive products is exemplified by the following:

(a) The beta-blocker, propranolol, of the chemical class, aryloxypropanolamine (Figure 27);
(b) The β-hydroxy β-methylglutaryl coenzyme A reductase inhibitor, atorvastatin (used in lowering blood cholesterol level; Figure 28).

Metabolic-ring hydroxylation leading to enhancement of activity is exemplified by acetanilide to paracetamol, which was already discussed in Section 5.1.1.

#### 5.3.1. Metabolic Aromatic-Ring Hydroxylation Leading to Loss of Activity

##### Phenobarbital/Phenytoin

The anticonvulsant drug, phenobarbital (Figure 18), was chosen as a representative of the barbiturate group of drugs because it is the only member that contains an aromatic benzene ring susceptible to metabolic hydroxylation. On the other hand, the anticonvulsant drug, phenytoin (Figure 18), was selected for its similarity to phenobarbital with respect to chemical structure, pharmacologic activity, and even mechanism of action. Phenytoin additionally contains two benzene rings with an identical chemical environment. Both phenobarbital and phenytoin are metabolized by aromatic-ring hydroxylation (Figure 18), a process that led to a loss of pharmacologic activity [64,65,66,67].

The CNS depressant activity of barbiturates (sedative, hypnotic, and anticonvulsant) and its termination are mainly dependent on the drug lipophilicity, which is imparted by the aromatic rings and the alkyl groups [68]. Lipophilicity helps the barbiturates to cross the blood–brain barrier, exert their effects, and again facilitate their distribution to other tissues, thus reducing their effective concentrations at the brain’s gamma-aminobutyric acid receptors (GABA receptors). This redistribution process of the barbiturates is considered a deactivation process. In addition, metabolism plays a role in the deactivation of barbiturates. All barbiturates contain two alkyl groups at carbon 5 of the barbituric acid ring (Figure 18) with the exception of phenobarbital, which contains an alkyl group (ethyl) and a phenyl group. All the alkyl groups in barbiturates are metabolized by oxidation in phase I at the ω or ω-1 carbons to either primary or secondary alcohols, respectively. The primary alcoholic groups may further be oxidized to carboxyl (COOH) groups. On the other hand, the phenyl group in phenobarbital is metabolically oxidized to a phenolic group at the favored *para* position (Figure 18). All such hydrophilic functionalities are detrimental to the essential hydrophobicity of the alkyl and phenyl groups, and thus, to the ability of the resulting metabolites to cross the blood–brain barrier. Enhancement of the water solubility of the barbiturate metabolites and their subsequent, fast elimination is a further cause of termination of pharmacologic activity resulting from the introduction of hydrophilic functionalities. Even more, the phase I metabolites, either carboxylic or phenolic, may further be conjugated in phase II to glucuronides and/or sulfates (Figure 18) with a consequent further increase in water solubility, elimination, and termination of activity due to a reduction in the effective concentration at the receptor. 

Despite the fact that the pharmacokinetics of the barbiturates play a major role in their deactivation, a pharmacodynamic dimension cannot be excluded: the introduction of a hydrogen-bonding functionality, such as the hydroxy (OH) group, on an essentially hydrophobic site will most probably negatively affect binding to the GABA_A_ receptor, resulting in a loss of pharmacologic activity. Furthermore, by replacing a hydrogen atom in the benzene ring, the hydroxyl group will create a steric effect and increase the metabolite molecule size, factors that are detrimental to the proper metabolite–receptor interactions, and thus, to a loss of pharmacologic activity.

The two aromatic rings in phenytoin have identical chemical environments, and only one of them is hydroxylated, which is consistent with the rules of metabolic aromatic-ring hydroxylation. The same reasoning that applies to the loss of activity of the phenolic metabolite of phenobarbital discussed above should apply to the loss of activity of the phenolic metabolite of phenytoin. Further reasoning is considered in the discussion section.

##### Benzodiazepines: Diazepam and Estazolam

The benzodiazepines are CNS depressants used as minor tranquilizers, sedatives, hypnotics, and anticonvulsants; in these respects, they largely supersede the barbiturates. Their mechanism of action involves binding to GABA_A_ receptors [69,70,71]. The benzodiazepines are metabolized through several phase I oxidative reactions, with some followed by phase II conjugative reactions. The metabolic pathways of the two benzodiazepine representative members, diazepam [72,73] and estazolam [74,75], are shown Figure 19 and Figure 20, respectively, with only aromatic-ring hydroxylation discussed in this section. The other metabolic pathways of diazepam and estazolam are discussed where relevant. 

Due to favorable electronic and steric structural environments, diazepam and estazolam are about the only two members of the clinically used benzodiazepines to undergo metabolic aromatic-ring hydroxylation at the 4′ position (Figure 19 and Figure 20). In most of the other members, metabolic 4′-hydroxylation is possibly disfavored by the presence of an electron-withdrawing halo group at position 2′ (fluoro in flurazepam, flunitrazepam, and quazepam, and chloro in triazolam and clonazepam; for the numbering of the benzodiazepine ring system, reference should be made to the structure of diazepam in Figure 19). 

The 4’-hydroxylation of both diazepam [72,73] and estazolam [74,75] resulted in inactive metabolites. Foye (2013) attributed the loss of sedative, hypnotic effects of 4’-hydroxyestazolam to two factors [71]. The first factor is of a pharmacodynamic nature. It is explained by the 4’-hydroxy group weakening optimal binding of the aromatic ring to the GABA_A_ receptor via a steric effect: the hydroxyl group is substantially bulkier than the hydrogen atom. The expected result is, therefore, decreased receptor affinity and drug potency. The second factor is of a pharmacokinetic disposition, and results from decreased hydrophobicity (i.e., increased hydrophilicity), which results in the decrease of the effective concentrations of the circulating 4′-hydroxy metabolites due to enhanced polarity, water solubility, and elimination, as per se and as glucuronide conjugates. In the absence of reports regarding the loss of activity of 4’-hydroxydiazepam, an analogy may be extrapolated from that of 4′-hydroxyestazolam. 

In contrast to 4’ hydroxylation, metabolic hydroxylation at position 3 of the diazepine ring in diazepam (Figure 19) does not affected activity; rather, it introduces a pharmacokinetic dimension: the 3-hydroxy metabolite is more hydrophilic and is subject to glucuronide conjugation with subsequent enhanced rate of elimination, and hence, a shorter duration of action than diazepam. These observations tend to consolidate a major pharmacodynamic role of the 4’-aromatic-ring hydroxyl group on causing loss of activity of diazepam, as well as of estazolam. The plausible explanation is that the introduction of the hydrogen-bonding group (the hydroxy) into an essentially pharmacophoric hydrophobic moiety (the benzene ring) is detrimental to the optimal GABA_A_ receptor binding.

A halo group at position 2’ of the benzodiazepine backbone (ring C, Figure 19) serves three purposes: it adds a welcomed hydrophobicity to the drug, disfavors metabolic ring hydroxylation, and imparts a conformation-locking effect on the aromatic ring through a steric effect. The consequence of these effects could be enhanced selectivity on drug–receptor interaction, leading to higher efficacy and possibly higher potency. Increased hydrophobicity would tend to enhance blood–brain barrier penetration, and therefore, increased access to the GABA_A_ receptor.

In the above context, it would be worthwhile to investigate the effect of another halo group at position 6’ (Figure 19) on the conformation locking of the aromatic rings in analogy with the NSAID pair, fenoprofen/diclofenac. In diclofenac (Figure 21), the two *ortho*-positioned chloro groups resulted in a restricted conformation with consequent enhanced selectivity, efficacy, and potency compared to fenoprofen, in which the two chloro groups are absent [76].

With respect to stereochemistry, both diazepam and estazolam are achiral. However, metabolic hydroxylation at C3 of diazepam and C4 of estazolam (Figure 19 and Figure 20, respectively) results in chiral metabolites. The significance of such chirality as related to metabolite activity is discussed in Part 2 of this review series, which deals with metabolic aliphatic-ring hydroxylation. 

##### NSAIDs

The chemical classes of NSAIDs were discussed in Section 5.1.3.

(1) Diclofenac

Diclofenac is a phenylacetic acid NSAID. It was developed as a variant of fenoprofen by introducing two *ortho*-positioned chloro groups in the anilino-aromatic ring to restrict its free rotation [76]. This restriction of rotation increases selectivity, and hence, potency with respect to fenoprofen. The metabolism of diclofenac shown in Figure 21 [77] represents one of the anomalies of aromatic-ring hydroxylation in that hydroxylation occurs at the *meta* position of two chloro groups. The hydroxy metabolites are pharmacologically inactive. 

In accounting for the structure–activity relationship of diclofenac, Foye (2013) [78] proposed that the function of the two *ortho* chloro groups was to force the anilino-phenyl ring out of the plane of the phenylacetic acid portion. Such twisting, as proposed by Foye (2013) [78], is important in the binding of diclofenac to the active site of COX. The introduction of a hydroxy group in the anilino-phenyl group would create a hydrogen-bonding characteristic, which would weaken or hinder the necessary twisting, thus resulting in an attenuation or loss of pharmacologic activity.

(2) Ketorolac

Ketorolac (Figure 22) is a pyrrole-acetic acid derivative structurally related to indomethacin and tolmetin. It is metabolized to *p*-hydroxyketorolac (Figure 22), which is inactive. Both the carboxy and phenolic hydroxy groups are further metabolized via glucuronide conjugation to give pharmacologically inactive products [79,80]. In analogy to the previously discussed cases, the loss of activity of 4-hydroxy ketorolac may be attributed to both pharmacodynamic and pharmacokinetic effects. Pharmacodynamic effects are elaborated upon in the discussion section.

Ketorolac contains a chiral center as designated by the asterisk in Figure 22. Its enantiomers differ in their pharmacodynamic effect: while the *S-*enantiomer acts as both a COX1/COX2 inhibitor and an analgesic, the *R*-enantiomer only retains the analgesic activity [80]. The stereochemical basis of the loss of activity caused by metabolic aromatic-ring hydroxylation of ketorolac is not known.

(3) Flurbiprofen

Flurbiprofen is an arylpropionic acid COX1/COX2-inhibitor NSAID. It is mainly metabolized by aromatic-ring hydroxylation, as shown in Figure 23, with loss of activity [81]. The metabolism of flurbiprofen shows a rather interesting pattern in that a catechol ring is formed in which the 4′-position is anomalously methylated to yield a methoxy group with reduced polarity. This metabolic route is reminiscent of that of adrenaline, which is metabolized by the methylation of the *para*-hydroxy group by the enzyme catechol-*O*-methyl transferase (COMT) [82]. Furthermore, the metabolic double hydroxylation of flurbiprofen to yield 3’,4’-dihydroxy flurbiprofen is also an anomaly of metabolic-ring hydroxylation. We recall metabolic aromatic-ring dihydroxylation in the same molecule is generally disfavored [65,66]. Similar to naproxen, flurbiprofen interacts with the COX active site through ion pairing involving the carboxylate group and van der Waals contacts involving the α-methyl and phenyl groups. The metabolically introduced hydroxyl groups on the aromatic ring will be detrimental to the hydrophobic-pocket fit, thus leading to a loss of activity. Other factors are considered in the discussion section. 

Being an arylpropionic acid derivative, flurbiprofen is a chiral drug as indicated by the asterisk in Figure 23. As for all members of the arylpropionic acid NSAID subclass, the anti-inflammatory (COX-inhibiting) activity resides in *S*-(+)-flurbiprofen; *R*-(−)-flurbiprofen is expected to be the enantiomer with analgesic activity. Whether metabolic aromatic hydroxylation of flurbiprofen abolishes both anti-inflammatory and analgesic activities or exhibits selectivity is not known.

##### Miscellaneous: Warfarin

Warfarin (Figure 25) is an anticoagulant drug used as a prophylactic in preventing thrombus formation in patients who are at high risk of developing thromboembolic disease. Warfarin is chiral with the *S*-enantiomer having three- to five-fold the activity of the *R*-enantiomer [83]. Warfarin contains two aromatic moieties, a benzopyran and a phenyl, in addition to a 2-butanone chain (Figure 25). The major metabolic pathway of warfarin is through the hydroxylation of the benzene ring of the benzopyran moiety, in addition to a minor route through the reduction of the side-chain keto group to a secondary alcohol (Figure 24) [84,85,86,87]. *S*-warfarin is metabolized by the CYP2C9 isoenzyme to *S*-7-hydroxywarfarin, while *R*-warfarin is metabolized by CYP1A2, CYP3A4, and CYP2C19 isoenzymes to *R*-6, *R*-7, *R*-8, and *R*-10 hydroxy warfarins [88]. The acidic hydroxy group at C4, being in conjugation with the benzene ring, possibly explains the preference of metabolic hydroxylation of the benzopyran ring over the phenyl group since it increases the electron density toward hydroxylation through a positive inductive effect. Furthermore, both warfarin enantiomers are metabolized through reduction of the side-chain keto group by carbonyl reductase to a secondary alcohol (Figure 25) [85,86,87,88]. It was observed that the hydroxy group introduced metabolically on the aromatic ring led to a loss of anticoagulant activity, while the hydroxy group resulting from side-chain keto reduction resulted only in attenuation of activity [85]. In addition, the metabolic reduction of the side-chain keto group in warfarin created a second chiral center. Such a state of affairs may impact on the pharmacodynamic and pharmacokinetic effects of the 11-hydroxywarfarin metabolite (Figure 25). Keto-group reduction and pharmacologic activity are the subject of alcoholic metabolites to be discussed in Part 2 of this review series. 

#### 5.3.2. Metabolic Aromatic-Ring Hydroxylation Resulting in Attenuation of Pharmacologic Activity

##### Chlorpromazine

Chlorpromazine (Figure 26) is a major tranquilizer used as an antipsychotic. It is metabolized in humans via two major routes [89,90,91,92,93]: (a) aromatic-ring hydroxylation at position 7 to a moderately active metabolite (Figure 26); and (b) sulfoxidation to an inactive metabolite. A minor deactivating route through *N*-demethylation also occurs. It should be noted that the metabolic hydroxylation of chlorpromazine is in accordance with the rule that groups with negative inductive effects, such as chloro, deactivating the ring to hydroxylation.

#### 5.3.3. Aromatic-Ring Hydroxylation Resulting in Parent-Drug Equiactive Metabolites

##### Propranolol

Propranolol (Figure 27) is an aryloxypropanolamine β-adrenoceptor blocker used as an antihypertensive and antiangina agent. As shown in Figure 26, it is metabolized in humans to three major metabolites, two of which are inactive and one of which is as active as the parent drug. In naphthoyloxyacetic acid (metabolite I, Figure 27), the pharmacophore is ruptured, leading to a loss of activity. In metabolite II, glucuronide conjugation of the side-chain hydroxy group led to a loss of activity, while in 4-hydroxypropranolol (metabolite III), activity was maintained. Furthermore, 4-hydroxypropranolol is metabolized in phase II to the inactive glucuronide conjugate [94,95]. The metabolism of propranolol directs attention to two interesting points: (a) glucuronic-acid conjugation of both alcoholic and phenolic hydroxy groups leads to a loss of pharmacologic activity, a phenomenon that is true for most cases; and (b) alkoxy groups are aromatic-ring activators and *para*-directors in metabolic aromatic-ring hydroxylation. 

Propranolol is a monochiral drug as indicated by the asterisk in Figure 27. For all the monochiral aryloxypropanolamines, the β-adrenoceptor-blocking activity resides mainly in the *S*-(−)-enantiomer, with the *R*-(+)-enantiomer exhibiting minimal activity [96]. Despite this fact, however, all the clinically used aryloxypropanolamine β-adrenoceptor-blockers, except *S*-(−)-timolol, are used as racemates. When the metabolic change occurs at a group distant from the chiral center, as in 4-hydroxypropranolol, extrapolation of the absolute enantiomeric designation from the parent drug to the metabolite is possible. Accordingly, 4-hydroxypropranolol may be assigned the *S* designation. However, optical activity designation and pharmacological activity may not be extrapolated from the parent drug and are only subjects of experimental verification. 

##### Atorvastatin

Atorvastatin (Figure 28) is an HMG-CoA reductase inhibitor used in lowering blood cholesterol and triglyceride levels. Atorvastatin is metabolized by CYP3A4 hydroxylation at the *ortho*- or *para*-position at ring D, as shown in Figure 28 [97,98]. Being electron withdrawing, both the fluoro group and the pyrrole ring (C) disfavor metabolic hydroxylation of rings A and B, respectively. Accordingly, in accordance with the rule, hydroxylation takes place at the least hindered benzene ring, i.e., ring D. The two metabolites are equiactive with the parent drug and account for about 70% of its overall circulating activity [97,98]. As designated by the asterisks in Figure 28, atorvastatin is a bichiral drug, and hence, exists in four enantiomeric forms: 3*R*5*R*, 3*R*5*S*, 3*S*5*R*, and 3*S*5*S* [99]. The marketed enantiomer is 3*R*5*S* [100]. The hydroxy metabolites of atorvastatin exist in the same enantiomeric forms as the parent drug.

In explaining the equiactivity of the atorvastatin hydroxy metabolites, the molecule of atorvastatin can be dissected into two parts: the dihydroxyheptanoic acid moiety and the aromatic ring system with its substituents. Dr. Philip Portoghese (1988) [101], a medicinal chemist from the University of Minnesota, developed a concept called “message address,” which conceptually breaks a drug molecule up into two components: one, which “finds” the active site (the address), and the other, which actually delivers the drug’s chemical message. In atorvastatin, the dihydroxyheptanoic-acid moiety represents the message, while the aromatic-ring system with substituents represents the address. When the address is substantially large, a small-group metabolic change is not expected to result in a significant impact on its role. This is true for atorvastatin, which contains a four-ring system that mainly interacts with the active site of the enzyme via hydrophobic binding. 

Since the term “pharmacophore” is mostly used in the pharmacodynamics of drug action, we propose an adaptation of Dr. Portoghese’s concept by using the terms “primary pharmacophore” and “auxiliary (logistic) pharmacophore” as equivalent terms to “message” and “address”, respectively. By binding to the receptor, the auxiliary (logistic) pharmacophore will facilitate the anchoring of the primary pharmacophore in the proper orientation in the receptor or enzyme active cavity. The primary pharmacophore will then compete with the physiologic substrate for the binding sites in the receptor or enzyme active site. 

##### Phenylbutazone

Two metabolites of phenylbutazone (Figure 24), which were isolated from human urine, possess some of the pharmacological activities of the parent drug. Metabolite I (oxyphenbutazone), formed by aromatic-ring hydroxylation, has the potent antirheumatic and sodium-retaining effects of phenylbutazone; it was developed into a drug of its own right. On the other hand, metabolite II, formed by the oxidation of the ω-1 carbon of the butyl side chain, also possesses reduced sodium-retaining properties, but it is a considerably more potent uricosuric agent than phenylbutazone [102,103].

Phenylbutazone binds to and deactivates prostaglandin H synthase and prostacyclin synthase through peroxide (H_2_O_2_)-mediated deactivation. The reduced production of prostaglandin leads to reduced inflammation in the surrounding tissues [103]. It is also pertinent to note that γ-hydroxyphenylbutazone, which results from ω-1 hydroxylation of the butyl side chain in phenylbutazone, is devoid of anti-inflammatory activity [104]. Metabolic, aliphatic hydroxylation and pharmacologic activity of the resulting metabolites is the subject of Part 2 of this review series. 

Despite phenylbutazone being a monochiral drug, the relationship between its stereochemistry and activity does not receive much interest, possibly because it was developed and used at a time when stereoselectivity of drug action was not a well-developed science. 

### 5.4. Discussion of Metabolic Aromatic-Ring Hydroxylation

For the drug cases reviewed in this section, except for diclofenac, the structural features, either electronic or steric, set above for the occurrence of aromatic-ring hydroxylation, conform well to the rule of thumb.

Loss, decrease, or retention of pharmacologic activity upon aromatic-ring hydroxylation in the cited cases may reflect the status of the ring in the parent drug regarding its mechanism of interaction with the receptor. When hydrophobic binding of the aromatic ring with the receptor is essential for activity, introduction of the hydrophilic hydrogen-bonding hydroxy group will compromise the fit and will not be tolerated. The established hydrogen bonding may force the ring out of the plane of interaction with the receptor; the result will be loss of activity. The aromatic-ring dislodging is also assisted by a steric effect caused by the bulkier hydroxyl group in the metabolite relevant to the hydrogen atom in the parent drug. Furthermore, an increase in size and surface area of the metabolite caused by the hydroxyl group relevant to the parent drug may be synergistic factors in the poor fit of the aromatic ring in its hydrophobic pocket in the receptor. This was the case with phenobarbital and phenytoin (Figure 18), diazepam, (Figure 19), estazolam (Figure 20), NSAIDs (Figure 21, Figure 22 and Figure 23), and warfarin (Figure 25). Furthermore, the phase II glucuronide conjugation of the aromatic hydroxy group is an important factor in causing loss of activity of the metabolite. It considerably enhances metabolite clearance, and accordingly, reduces its effective concentration at the receptor or enzyme active cavity.

When aromatic-ring hydroxylation results in decreased activity, as in 7-hydroxychlorpromazine (Figure 26), three inferences present themselves as possible explanations of the observation: (a) the hydroxylated ring is auxiliary pharmacophoric; (b) the hydroxy group results in an increase in the optimal molecular size in the metabolite relative to the parent drug; (c) the hydroxy group weakens optimal binding of the aromatic ring to the receptor via a steric effect relevant to the hydrogen atom. In addition, a reduction in the effective concentration of the hydroxy metabolite at the receptor through glucuronide conjugation may play an important role. 

**Figure 29:**
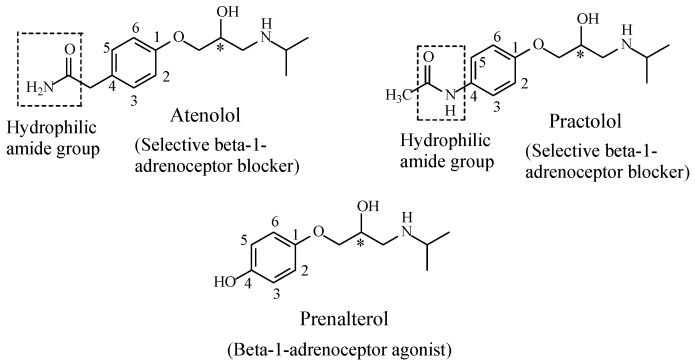
Structures of atenolol and practolol.

When the hydroxy metabolite is equiactive with the parent drug, two inferences can be tentatively made. Firstly, the hydroxylated aromatic ring is auxiliary, i.e., it plays the role of the address. This is the case with the statin drug, atorvastatin (Figure 28), where the three-aromatic-ring system is auxiliary pharmacophoric, and therefore, is not involved in essential binding to the enzyme [105]. However, the role of the three-aromatic-ring system is logistic, that of proper anchoring of the drug in the enzyme active cavity for optimal interaction of the primary pharmacophoric groups with the enzyme to take place. The second inference is associated with 4-hydroypropanolol (Figure 27), the equiactive metabolite of propranolol. Propranolol is a nonselective β1/β2-adrenoceptor blocker; it belongs to the aryloxypropanolamine chemical class. In this class of compounds, a hydrophilic amide substitution at position 4 of the aromatic ring imparts β1 antagonistic selectivity [106], such as in atenolol and practolol (Figure 29). On the other hand, a hydrophilic hydroxy substitution at the same position reverses the activity altogether, i.e., from antagonistic to agonistic, such as in prenalterol (Figure 29) [107]. It can, hence, be concluded that the nature of the hydrophilic group substitution at position 4 of the aryloxypropanolamines significantly dictates the pharmacologic outcome of the β1-receptor interaction. Based on the above facts, it may be inferred that 4-hydroxypropranolol is a tentative β1-adrenoceptor agonist pending experimental verification.

The atorvastatin equiactive hydroxy metabolites furnish a useful inference: pharmacologic equiactivity of metabolites relative to the parent drug occurs when the metabolic functionalization takes place at the “address” or “auxiliary pharmacophore”. We will provide further examples of this phenomenon in Part 2 of this review series.

Of the NSAIDs, phenylbutazone stands in a class of its own in that its mechanism of action does not involve the inhibition of COX, but rather the inhibition of prostaglandin H synthase. As shown in this section and in Section 5.1.3, a hydroxy group on the aromatic rings of arylalkanoic-acid COX1/COX2-inhibitor NSAIDs is detrimental to their pharmacologic activity. This is in contrast to phenylbutazone whose aromatic-hydroxy metabolite (oxyphenbutazone, Figure 24) is equiactive with the parent drug and was developed into an anti-inflammatory drug of its own right. The different mechanisms of action, and accordingly, the varying sites of drug action involved may explain the disparity between the activities of the hydroxyl metabolites of phenylbutazone and the arylalkanoic-acid NSAIDs.

While pharmacodynamics may handsomely explain the pharmacological activity of drug hydroxy metabolites relative to the parent drugs, the role of the pharmacokinetics of these metabolites should not be excluded. In the cases where the pharmacologic activity of the hydroxy metabolite is either attenuated or lost relative to the parent drug, pharmacokinetic factors may come into perspective in two aspects. Firstly, the hydroxy metabolites are rapidly cleared by phase II conjugation, thus aiding in the termination of their action. Secondly, the hydroxy metabolites do not readily penetrate target tissues due to a reduction in membrane permeability caused by an increase in polar surface area, a limitation that affects their active concentrations at the target site [108].

## 6. Racemic Drugs versus Enantiopure Drugs

Of all the chiral drugs presented as examples in this review, only levorphanol, *S*-(+)-naproxen, and 3*R*5*S*-atorvastatin are used clinically in enantiopure forms; the rest of the drugs are used as racemates. The use of a chiral drug as a racemate does not necessarily imply that the constituent enantiomers are identical in their pharmacodynamic, pharmacokinetic, or toxicologic properties. Difficulties in preparing the pure enantiomers via the separation from racemates or via enantioselective synthesis may be prohibitive factors due to a lack of technology and/or high cost. In some instances, however, despite the feasibility of separating or synthesizing the eutomer of a chiral drug, big international pharmaceutical companies seem not to be interested in the practice and they keep to the racemates. Certainly, they have their undeclared reasons for this. Nonetheless, the differences between the pharmacologic properties of the enantiomers of a chiral drug may seem insignificant to warrant their costly separation from the racemates or stereoselective synthesis. *S*-(+)-naproxen represents an example of chiral-switch drugs. A chiral-switch drug may be defined as “an enantiopure drug whose racemate was once used or is currently used clinically. On the other hand, levorphanol and 3*R*5*S*-atorvastatin represent examples of what may be described as “from-onset enantiopure drugs”. Some scientists [109], though acknowledging the importance of enantiopure drugs in advancing pharmacotherapy, expressed their reservations on the production and use of chiral-switch drugs such as levocetirizine, esomeprazole, and escitalopram on the basis of the insignificant therapeutic advantages they present. To the list, we may add *S*-(+)-ibuprofen, (a chiral-switch drug marketed in some countries as dexibuprofen). The addition is made on the basis of the metabolic inversion of the inactive *R*-(−)-enantiomer to the active antipode, as well as the current marketing of the racemate by big international pharmaceutical companies. 

### Glucuronide Conjugation: Prevalence and Effect on Pharmacologic Activity

Glucuronide conjugation is the most common phase II metabolic process [110], though certain structural features control its occurrence. It occurs with almost all aromatic hydroxy (arenolic) groups, most carboxyl groups, unhindered alcoholic hydroxy groups, and a few amino and sulfhydryl groups. Because of its relatively big size, glucuronic acid may not have easy access to active hydrogen-containing groups that are awkwardly situated in a molecule, i.e., sterically hindered groups. For instance, tertiary alcoholic groups such as 14-hydroxy in oxycodone (Figure 5) and 1′-hydroxy in tramadol (Figure 6) are sterically hindered, and hence, are not susceptible to glucuronide conjugation. 

With the exception of codeine glucuronide and morphine-6-glucuronide, glucuronide conjugation led to a loss of activity in all the cases presented in this review. With three hydroxy moieties and a completely ionized carboxyl moiety at physiologic pH, the glucuronide group considerably increases metabolite hydrophilicity, water solubility, elimination, and termination of action. In the end, the termination of activity is a consequence of the reduction in the effective concentration of the glucuronide conjugate at the receptor. With this pharmacokinetic concept of loss of activity for most drug glucuronide conjugates, the door is open only to pharmacodynamic speculation to explain the activity of codeine glucuronide and morphine-6-glucuronide. 

## 7. Conclusions

The hydroxy group is the most common hydrophilic group produced by metabolic functionalization in drug molecules. Aromatic hydroxy groups result in one of two ways: the *O*-dealkylation of aralkoxy groups or the hydroxylation of the aromatic ring. The *O*-dealkylation of aralkoxy groups in drug molecules is an invariably predictable metabolic route. On the other hand, metabolic aromatic-ring hydroxylation is governed by electronic and steric factors prevailing in the ring. The pharmacologic activity of the resulting arenolic metabolites resulting from both processes depends on the site of drug action (i.e., the receptor), as well as on the pharmacophoric or auxophoric status of the aromatic ring to which the alkoxy or hydroxy group is bonded. In general terms, phase I metabolic functionalization may reveal the status of a group in a drug molecule, whether primary or auxiliary pharmacophoric or auxophoric. Moreover, if there is previous knowledge of the pharmacophoric or auxophoric statuses of the rings, then the effect of the metabolically formed hydroxy group on the activity of the metabolite may be predicted. In addition to pharmacodynamic effects, the attenuation or loss of activity of polar metabolites may be explained by pharmacokinetic effects, which, by enhancing elimination, lead to a reduction in metabolite effective concentration at the receptor. 

When more active or equiactive metabolites showed favorable pharmacodynamic and pharmacokinetic properties, they were developed into drugs of their own rights. Nevertheless, not all equiactive metabolites were developed into drugs of their own rights, and hence, may be classified as drug-action-extension forms.

Since, for the chiral drugs presented in this review, metabolic functionalization occurred at sites distant from the chiral center, absolute enantiomer configuration (as *R* or *S*) may be extrapolated from the parent drug to the hydroxy metabolite. However, optical activity designations (*dextro* or *levo*), as well as pharmacological activity, are the subject of experimental verification.
